# Promoting Oral Health for Patients with Special Needs

**DOI:** 10.3390/ijerph20136232

**Published:** 2023-06-27

**Authors:** Frédéric Denis, Hervé Becquet, Matthieu Renaud, Guillaume Savard

**Affiliations:** 1 Faculty of Dentistry, Tours University, 37000 Tours, France; matthieu.renaud@univ-tours.fr (M.R.); guillaume.savard@univ-tours.fr (G.S.); 2 Department of Odontology, Tours University Hospital, 37000 Tours, France; 3 EA 75-05 Education, Ethique, Santé, Faculté de Médecine, Université François-Rabelais, 37000 Tours, France; 4 Department of Odontology, Orleans Hospital, 45100 Orleans, France; herve.becquet@univ-tours.fr

The problem of poor oral health among people with disabilities is common in many low-, middle- and high-income countries [1]. An estimated 1.3 billion people, or one in six of the world’s population, are disabled [2]. Improving the oral health of this population is, therefore, a major public health challenge. Against this backdrop, health policies must invest in this field of health in a convincing and evidence-based way.

One of the challenges of this Special Issue titled “Promoting oral health for patients with special needs” is to help understand the complex relationship between oral health problems and disability. It is often the case that an aspect of disability, initially associated with a specific condition, is in fact linked to concomitant problems [3]. The coexistence of multiple health problems can also make it more difficult to manage health services [4]. It is, therefore, necessary to highlight all initiatives aimed at promoting ways of improving the oral health of people with disabilities, whether in health promotion and prevention, improving access to care, training health professionals and carers, or in the field of clinical and/or basic research.

Indeed, the consequences of poor oral health are numerous, not least of which are seen in one’s quality of life, with painful episodes caused by untreated carious lesions [5]. People with disabilities often delay seeking care because they do not have sufficient support or an adaptive care program. For example, in the case of people suffering from psychosis, the symptoms caused by dental pain may be associated with decompensation of the psychiatric illness, leading to a difficult diagnosis and a delayed return to care. Delayed treatment often leads to more radical care, i.e., extraction of the dental organ, as in some cases, the dental tissue is too altered to allow for restoration ad integrum [6]. If these extractions are not compensated for, they lead to a loss of masticatory function and physical integrity, which are detrimental to one’s self-esteem.

People with disabilities must be able to benefit from quality healthcare services and professionals trained to meet their specific needs on an equal footing with others, even in times of health crises. This was evident in studies carried out during the pandemic, which showed that poor periodontal health increased the risk of COVID-19 [7,8]. People with disabilities are twice as likely to develop disorders and diseases, such as depression, asthma, diabetes, stroke, obesity, and poor oral health [9]. Although the oral health status of the population has improved in much of the world, patients who are disabled remain at a disadvantage in all countries. In many high-income countries, providers use specialized approaches, increasingly dominated by high-tech treatments, but do not address the underlying causes of disease or inequalities in oral health [10]. Furthermore, in low- and middle-income countries, oral health care is often unavailable, unaffordable, and inappropriate for the majority of the population. As oral pathology shares common risk factors with many other diseases, an inter- and transdisciplinary approach that addresses common risk factors should be encouraged in the interest of efficiency, equity, and the maintenance of oral health.

In the field of basic research, certain studies on microbiota point to promising therapeutic avenues for improving oral health and, more generally, the overall health of people with disabilities. In the case of Alzheimer’s disease and late cognitive impairment, it is now accepted that targeting a novel oral fragility phenotype to maintain or even improve oral function and nutritional status could reduce the burden of oral dysfunction. The oral microbiota may influence the risk of Alzheimer’s disease through circulatory or neural access to the brain and through interaction with periodontal disease, which often leads to tooth loss. Periodontal disease is also linked to an increased risk of Alzheimer’s [11] and other systemic diseases, such as diabetes, rheumatoid arthritis, and systemic lupus erythematosus, which increase susceptibility to destructive periodontal disease [12].

Although dental care is not available remotely, digital health could facilitate early detection and the monitoring of diseases and conditions, and direct patients to primary care as quickly as possible [13]. 

Conclusions

The aim of this Special Issue is to highlight the health needs of people with disabilities, and to highlight convincing interventions in the field and/or areas of research likely to contribute to the reduction in health inequalities.
